# The packing fraction of the oxygen sublattice: its impact on the heat of mixing

**DOI:** 10.1007/s00269-024-01277-6

**Published:** 2024-06-04

**Authors:** Artur Benisek, Edgar Dachs

**Affiliations:** Chemistry and Physics of Materials, University of Salzburg, Jakob-Haringer-Str. 2a, 5020 Salzburg, Austria

**Keywords:** Interaction energy, Excess enthalpy of mixing, Stiffness, Oxygen packing density, Density functional theory

## Abstract

The heat of mixing of some petrological relevant substitutions (i.e., Mg-Al, Si-Al, Mg-Ti, Mg-Ca, and Mg-Fe) was investigated systematically in silicates, titanates, tungstates, carbonates, oxides, hydroxides, and sulphates by density functional theory calculations (e.g., melilite, chlorite, biotite, brucite, cordierite, amphibole, talc, pseudobrookite, pyroxene, olivine, wadsleyite, ilmenite, MgWO_4_, ringwoodite (spinel), perovskite, pyrope-grossular, magnesite-calcite, MgO-CaO, anhydrous and different hydrated MgSO_4_). A specific substitution is characterised by different microscopic interaction energies in different minerals, e.g., the octahedral Mg-Al exchange on a single crystallographic site in pyroxene has a microscopic interaction energy that is more than twice compared to that in biotite. A comparative investigation of the heat of mixing using microscopic interaction energies on a single crystallographic site has the advantage that they are not influenced by cation ordering. They could be successfully correlated with the stiffnesses of the minerals, which in turn were scaled to the oxygen packing fraction, a parameter that is easily available for poorly investigated minerals. With this information, the interaction energies of a certain substitution can be transferred from minerals where they are well-known to mineral groups where they are less- or unknown. Using the cross-site terms and the microscopic interaction energies, the macroscopic interaction energies of the coupled substitution, e.g., Mg + Si = Al + Al, of biotite and pyroxene were calculated, which are, however, affected by cation ordering and different degrees of local charge balance, for which appropriate models are necessary.

**Supplementary Information:**

The online version contains supplementary material available at 10.1007/s00269-024-01277-6.

## Introduction

To describe the heat of mixing (Δ*H*^mix^) more accurate, a decomposition of the macroscopic interaction energies into interaction energies between elements on different crystallographic sites has been proposed, termed the micro-ϕ approach (Powell et al. 2014). It was used by these authors to extend these micro-ϕ parameters from minerals where good data exist to minerals where no or little data exist without considering the physical differences between them. Such an extension is, however, questionable. The different polyhedron sizes of the substituted and substituent cations generate local strain heterogeneities and hence strain energy in the solid solution giving rise to the heat of mixing/disordering (e.g., Boffa Ballaran et al. 1998; Tarantino et al. 2003; Carpenter 2003). Strain was found to play an important role in generating heat of mixing in many studies (e.g., Davies and Navrotsky 1983; Geiger 2001, Urusov 2001). Furthermore, it is to be expected that the generated strain energy depends on the stiffness of the mineral (e.g., Urusov 2001; Tarantino et al. 2003). Interaction energy differences between minerals might thus be correlated to differences in mineral elasticity. In biotite, for example, the energetic effect of the Tschermak´s substitution is lower than half of that in pyroxene (Dachs and Benisek 2019; Benisek et al. 2007), although the difference in end-member volumes is similar in both minerals (Dachs and Benisek 2019; Etzel et al. 2007).

However, a systematic investigation of Δ*H*^mix^ versus mineral stiffness is difficult because cation ordering has also a significant influence on the interaction energies, especially when coupled substitutions are considered, where the degree of local charge balances complicates the situation (Vinograd et al. 2007; Benisek and Dachs 2020). The decomposition of the macroscopic interaction energies into interaction energies between elements on a single crystallographic site (microscopic interaction energies), however, allows a comparison excluding the effect of cation ordering and local charge balances. We present here results from density functional calculations on microscopic interaction energies for a number of petrological relevant substitutions and their dependence on mineral elasticity. It is a common practice in thermodynamic modelling that the exchange energies of a certain substitution are expected to be the same in different minerals (e.g., Powell et al. 2014). The aim of this study is to show, that such an approach may result in wrong interaction energies and to present a possible way to take the physical differences between minerals into account.

## Experimental methods

### Calculations using the density functional theory (DFT)

Quantum–mechanical calculations were based on the DFT plane wave pseudopotential approach implemented in the CASTEP code (Clark et al. 2005) included in the Materials Studio software from Biovia®. The calculations used the local density approximation (LDA) for the exchange–correlation functional (Ceperley and Alder 1980) and norm-conserving pseudopotentials to describe the core-valence interactions. Studying the Mg-Fe substitution, the LDA + U (2.5 eV) method and a ultrasoft potential was used. For the k-point sampling of the investigated unit cells, a Monkhorst–Pack grid (spacing of 0.04 Å^−1^) was used (Monkhorst and Pack 1976) and convergence was tested by performing calculations using a denser k-point grid. The structural relaxation was calculated by applying the BFGS algorithm (Pfrommer et al. 1997), where the convergence threshold for the force on an atom was 0.01 eV Å^−1^. The excess internal energy of mixing (Δ*E*^mix^) was calculated using the single defect method, which is a particularly effective way to calculate it using DFT methods (e.g., Sluiter and Kawazoe 2002). The single defect method utilises large supercells of a host end member by inserting a single substitutional defect (Li et al. 2014). Using the calculated energy and the mole fraction of the supercell in combination with the end member data, the exchange energy can be calculated. The obtained value represents Δ*E*^mix^ of the disordered state (Vinograd and Sluiter 2006; Vinograd and Winkler 2010).

The difference between Δ*E*^mix^ and Δ*H*^mix^ is given by the volume term *P* Δ*V*^mix^, where *P* is the pressure and Δ*V*^mix^ is the excess volume of mixing, which is typically less than 1 J/bar/mol. At a pressure of 0 Pa, where the results of this study are compiled, *P* Δ*V*^mix^ is zero and Δ*E*^mix^ = Δ*H*^mix^.

Unit cells with element substitutions involving different charges, i.e., the Mg–Al substitution, were generated by charging the unit cell itself. The energy calculations of charged unit cells can be corrected (Makov and Payne 1995), which was not feasible during this study because this correction is not implemented in the Materials Studio´s CASTEP code. However, as shown below, the results were verified by transforming the microscopic interaction energies into macroscopic ones, where no charged unit cells exist, and compared with independently derived DFT-calculated macroscopic interaction parameters.

Generally, the symmetry constraints of a unit cell that incorporates a single defect are destroyed generating a cell with *P1* symmetry. This local structural situation cannot be observed by X-ray diffraction because this technique averages over hundreds of unit cells and does not yield the correct crystal system of a given single unit cell. However, in a real crystal, a given unit cell cannot change its symmetry independently from their neighbouring cells. The unit cell with a defect will probably have a symmetry that is lowered compared to the end-member unit cell, but it will be higher than *P1* symmetry. These considerations were used to generate the crystal structure of the unit cell that contains a single defect.

The investigation focused on the octahedral Mg–Al substitution. The studied minerals are partly characterised by different octahedral sites. If the preferred site for Al is not known, the interaction energies were calculated for each octahedral site. In the case that one site is characterised by a significant lower interaction energy, ordering on this site was assumed (e.g., Al on M1 in olivine). On the other hand, if the energies are similar for different sites, they were combined into one single crystallographic site (e.g., Al on M1 + M2 in wadsleyite). Tab. 1 presents detailed information on the end-member choice and in consequence on the crystallographic site where mixing was assumed to take place (given in the supplementary material).

## Results

**Fig. 1:**
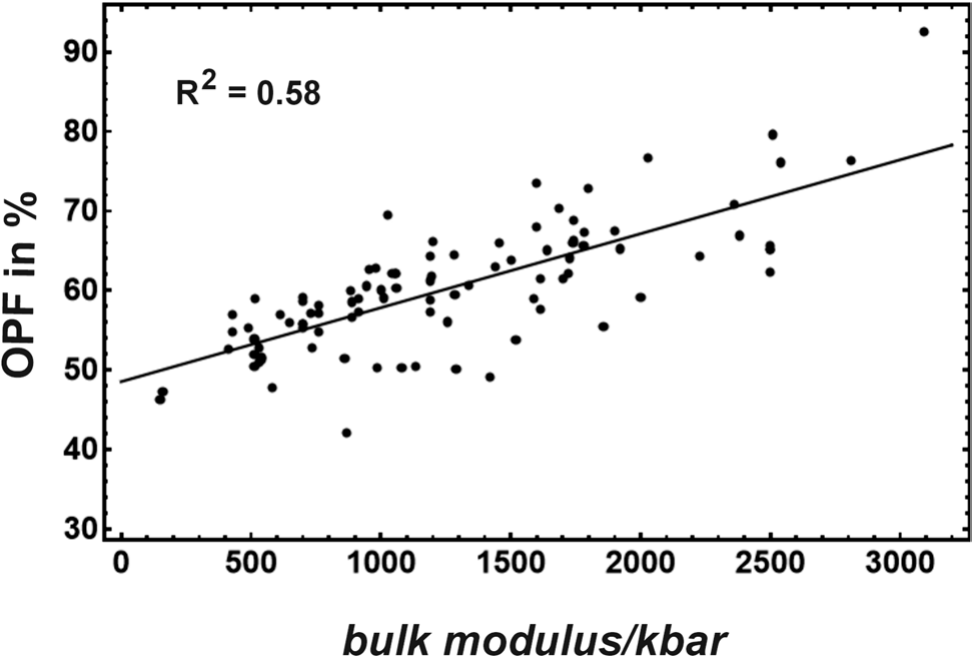
Oxygen packing fraction (OPF) plotted against bulk modulus for different minerals. Bulk modulus and volume data were taken from Holland and Powell (2011). The volume of the oxygen anion was calculated from the data of Shannon (1976)

To express the elastic properties of a mineral, the bulk modulus or compressibility is the mostly used parameter, however, for rare minerals it is not well known. During the search for another parameter, the question raised, which structural units determine the stiffness of a mineral. For minerals containing an oxygen sublattice, its packing fraction is likely to be a good proxy for the elastic properties since it dominates the occupancy of the unit cell volume. The oxygen packing fraction (OPF) is a parameter that has the great advantage that it is easily available for poor investigated minerals because OPF can be simply defined as

1 $${\text{OPF }} = \, n_O V_O /\left( {V_{UC} } \right),$$

where n_O_ is the number of oxygens in the unit cell, V_O_ is the volume of the oxygen anion calculated from the data of Shannon (1976), and V_UC_ is unit cell volume. The correlation between OPF and bulk modulus for different minerals is shown in Fig. 1 demonstrating that OPF increases from 51 to 71% during a bulk-modulus increase from 300 to 2500 kbar. The data are from the compilation of Holland and Powell (2011) and range from tridymite with low bulk modulus over all mineral groups that contain oxygen to stishovite with a high bulk modulus (including 95 minerals).

**Fig. 2:**
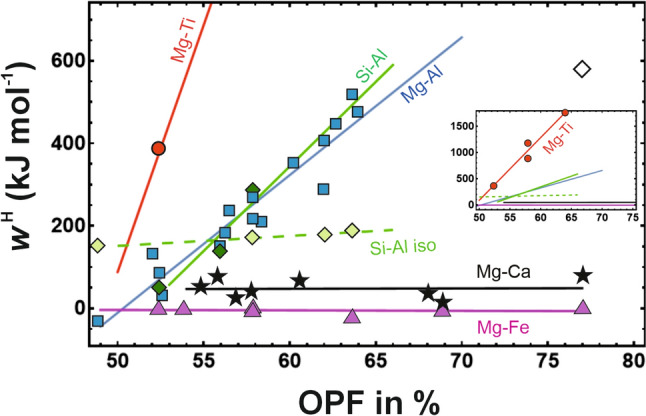
DFT calculated interaction parameters (*w*^H^) of different substitutions in different minerals as a function of DFT calculated oxygen packing fraction (OPF). The Mg-Al substitution is represented by a solid blue line and blue squares. The Si-Al substitutions are marked by a green solid line and green diamonds, the Si-Al substitutions with isolated substitution polyhedra by a broken green line and light green diamonds. The Mg-Ti^4+^ substitutions are marked by a red line and red circles, the Mg-Ca substitutions by a black line and black stars, and the Mg-Fe substitutions by a magenta line and magenta triangles. Open diamond represents the octahedral Si-Al substitution in MgSiO_3_ perovskite and is not included in the fit. Inset of the figure shows the whole Mg-Ti^4+^ substitution in relation to the other substitutions

Δ*H*^mix^ is defined as the difference between the enthalpy of a solid solution (*H*_AB_) at a given composition and the enthalpy of its mechanical mixture (*X*_A_ *H*_A_ + *X*_B_ *H*_B_), i.e.,

2 $$\Delta H^{{\text{mix}}} = H_{{\text{AB}}} - \, \left( {X_{\text{A}} H_{\text{A}} + X_{\text{B}} H_{\text{B}} } \right),$$

where *X*_A_, *X*_B_ and *H*_A,_ *H*_B_ represent the mole fractions and the enthalpies of the A and B component, respectively. Δ*H*^mix^ of different substitutions in different minerals was investigated by DFT using the single defect method (Sluiter and Kawazoe 2002). The data were then described by a symmetric Margules model,

3 $$\Delta H^{{\text{mix}}} = X_{\text{A}} X_{\text{B}} w^{\text{H}},$$

where *w*^H^ is the interaction energy between elements on a single crystallographic site (micro *w*^H^). This parameter is listed in Tab. 1 (given in the supplementary material) and plotted against OPF of the Mg-end members in Fig. 2.

The Mg-Al substitution was investigated in 16 minerals (ordered by increasing *w*^H^): melilite, chlorite, biotite, cordierite, amphibole, talc, pseudobrookite, pyroxene, sulphate (4 × hydrated), olivine, wadsleyite, sulphate (1 × hydrated), ilmenite, Mg-wolframite, anhydrous sulphate, and ringwoodite that has the spinel structure. As can be seen from Fig. 2, a linear positive empirical correlation of *w*^H^ versus OPF was found (fit parameters and R^2^ values of the correlation are given in the supplementary material). The Mg-Al substitution in biotite, as an example, has an energetic effect that is less than 1/2 of that in pyroxene, and less than 1/6 of that in spinel. This behaviour is expected to be a consequence of the different oxygen packing fractions, which are 53.5% in biotite, 59.1% in pyroxene, and 65% in spinel. In addition to the Mg-Al substitution, the Si-Al substitution has been investigated in 8 minerals. Here, two trends were observed. One, where the tetrahedra are connected with each other (chain and sheet silicates) investigated in 4 minerals (ordered by increasing *w*^H^): biotite, amphibole, pyroxene, perovskite. The second trend is from island and double island silicates defined by 4 minerals, i.e., melilite, olivine, wadsleyite, ringwoodite. This group is characterised by a much flatter positive correlation. MgSiO_3_ perovskite, with *w*^H^_SiAl_/OPF = 577.4/77.1, formed an outlier and was not included in the fit. The interaction energies of the Mg-Ca substitution were investigated in 7 minerals (ordered by increasing OPF): MgO-CaO solid solution, amphibole, olivine, pyroxene, carbonate, garnet, and perovskite). This substitution is characterised by a flat *w*^H^/OPF correlation. The interaction energies of this substitution are compared to experimental values from the literature (Kawasaki 1998; Liang and Schmid-Fetzer 2018; Newton et al. 1977; Vinograd et al. 2009) in Table 5 of the supplementary material. The Mg-Ti^4+^ substitution shows a steep slope that is defined by 4 minerals (ordered by increasing *w*^H^): biotite, pyroxene, olivine, ringwoodite. Finally, 7 minerals were studied for the Mg-Fe substitution finding ideal to slightly negative Δ*H*^mix^ behaviour (ordered by increasing OPF): biotite, brucite, pyroxene, olivine, spinel, garnet, and perovskite.

Using the interaction energies of the separate Mg-Al and Si-Al substitutions (micro *w*´s, i.e., *w*^H^_MgAl_ and *w*^H^_SiAl_) in combination with the underlying cross-site interaction (*w*^H^_cross_), the macroscopic interaction energy for the Tschermak´s substitution (*W*^H^_TS_) can be calculated (e.g., Powell et al. 2014):

4 $$W^{\text{H}}_{{\text{TS}}} = \, 1/4\,\left( {4\,w^{\text{H}}_{{\text{MgAl}}} + w^{\text{H}}_{{\text{SiAl}}} - \, 2\,w^{\text{H}}_{{\text{cross}}} } \right),$$

where the cross-site interaction is given by the energies of the reciprocal reaction:

5 $$w_{cross}^H = H\left( {Mg^{M} Si^{T} } \right) + H\left( {Al^{M} Al^{T} } \right) - H\left( {Mg^{M} Al^{T} } \right) - H\left( {Al^{M} Si^{T} } \right).$$

Such calculations were performed for the Mg–Al biotite in order to verify the DFT results of charged unit cells. Using Eq. (4), *w*^H^_MgAl_ = 82.5 kJ/mol, *w*^H^_SiAl_ = 95.6 kJ/mol, and *w*^H^_cross_ of 175.1 kJ/mol, the macroscopic interaction energy of *W*^H^_TS_ = 18.8 kJ/mol is obtained. It represents the interaction energy with ordering of Al on M1 and T1 sites, but without strict local-charge balance (without short-range ordering). To compare it with DFT values obtained without using charged unit cells, a Mg-biotite (KMg_3_[(OH)_2_AlSi_3_O_10_], phlogopite), a Tschermak substituted biotite (KMg_2_Al[(OH)_2_Al_2_Si_2_O_10_], eastonite) and intermediate solid-solution biotites were investigated. Using 10 different super cells for the intermediate biotites (50:50 composition with randomly distributed Al on M1 and T1 with sizes up to 16 formula units), a *W*^H^_TS_ of 19.0 ± 3.0 kJ/mol was obtained, which is in good agreement with the *W*^H^_TS_ calculated via micro *w*´s (18.8 kJ/mol). This value is slightly lower than an experimentally derived value of 25.4 kJ/mol (Dachs and Benisek 2019) because of different ordering schemes. The same calculations were performed for the diopside – CaTs solid solution, (*w*^H^_MgAl_ = 213.6 kJ/mol, *w*^H^_SiAl_ = 285.6 kJ/mol, and a *w*^H^_cross_ = 422.0 kJ/mol) yielding a *W*^H^_TS_ of 74.0 kJ/mol. The uncharged cells delivered 74.8 ± 3.7 kJ/mol, also in good agreement with *W*^H^_TS_ obtained via micro *w*´s. This value is larger than the experimentally derived value of ~ 24 kJ/mol (Benisek et al. 2007), if a symmetric fit is applied to their data. The difference comes mainly from the fact that the experimental data were derived using a partly disordered CaTs end-member (Benisek and Dachs 2020).

## Discussion

**Fig. 3:**
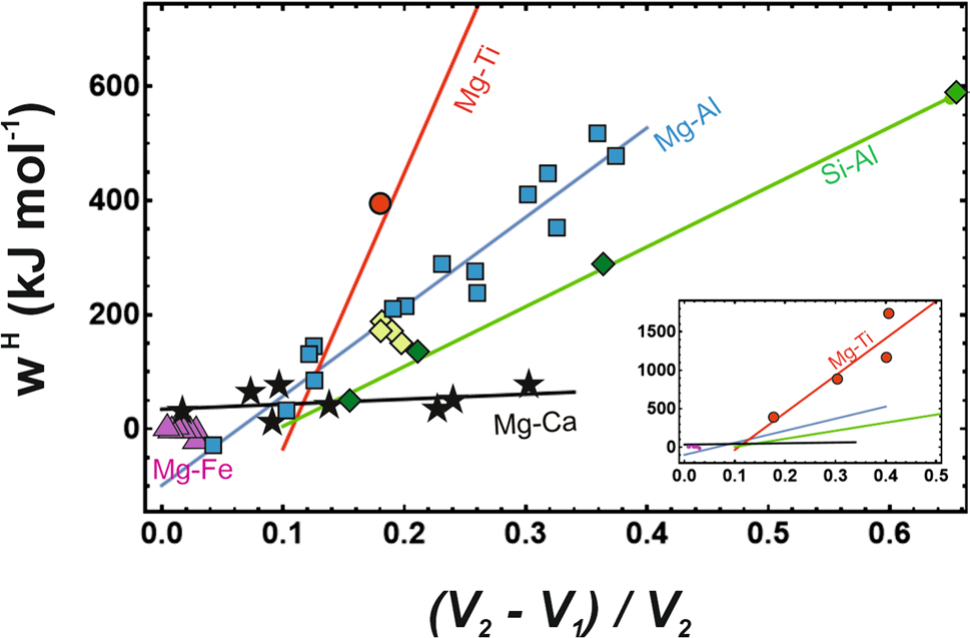
Interaction parameters (*w*^H^) of different substitutions and different minerals as a function of the DFT calculated normalised volume difference (V_2_–V_1_)/V_2_. The Mg-Al substitution is represented by a solid blue line and blue squares. The Si-Al substitution is marked by a green solid line and green diamonds. The island and double island silicates are characterised by a (V_2_–V_1_)/V_2_ of 0.17–0.19 and a *W*^H^ of ca. 170 kJ/mol marked by light green diamonds and are not included in the fit (solid green line). The Mg-Ti^4+^ substitutions are marked by a red line and red circles, the Mg-Ca substitutions by a black line and black stars, and the Mg-Fe substitutions by magenta triangles. Inset of the figure shows the whole Mg-Ti^4+^ substitution in relation to the other substitutions

Davies and Navrotsky (1983) correlated the interaction parameter (*W*) with a normalised volume difference (Δ*V*_norm_) as a quantity to include the strain, i.e.,

6 $$\Delta V_{{\text{norm}}} = \, \left( {{\text{V}}_2 -{\text{ V}}_1 } \right) \, /{\text{ V}}_2 ,$$

where V_2_ and V_1_ are the end-member volumes. The investigated minerals were also studied in this respect by the DFT methods, and the corresponding results are shown in Fig. 3 and compiled in Tab. 1 (fit parameter and R^2^ values are given in the supplementary material), demonstrating good correlations, if the different substitutions are separated.

**Fig. 4:**
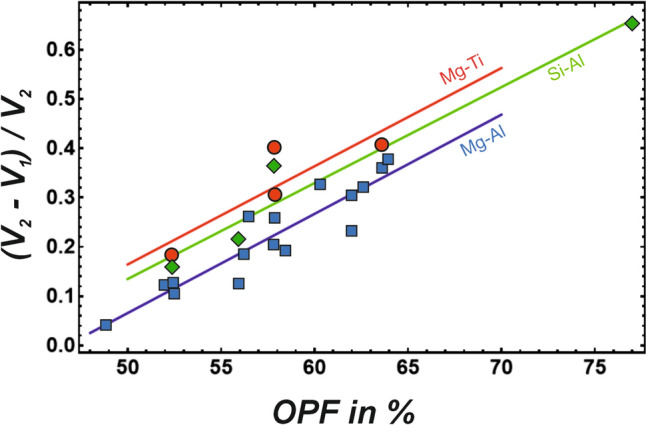
Normalised volume difference ((V_2_–V_1_)/V_2_) plotted against the oxygen packing fraction (OPF). The Mg-Al, Mg-Ti^4+^ and the Si-Al substitutions are represented by blue squares, red circles, and green diamonds, respectively

The *w*^H^/Δ*V*_norm_ and *w*^H^/OPF correlations yielded similar results, i.e., the Mg-Ti^4+^ has a steep and the Mg-Ca substitution has a very flat slope. They have also similar R-squared values (see supplementary material). However, the Si-Al substitution in MgSiO_3_ perovskite is no longer an outlier, as is the case for the *w*^H^/OPF correlation. From these points of view, there would be no need to postulate a relationship between *w*^H^ and OPF or between *w*^H^ and another parameter describing the mineral elasticity. However, Δ*V*_norm_ may also incorporate information on the packing density of the oxygen sublattice. To test this idea, Δ*V*_norm_ was plotted against the oxygen packing fraction (Fig. 4) demonstrating a good Δ*V*_norm_/OPF correlation.

Δ*V*_norm_, as used in the study of Davies and Navrotsky (1983) to parameterise the strain, contains thus also information about the packing density of the oxygen sublattice. For minerals with the same formula but different volumes (olivine versus ringwoodite), the Δ*V*_norm_/OPF correlation is easy to explain, because the volume of the ringwoodite is small compared to that of olivine increasing Δ*V*_norm_ from olivine to ringwoodite (from 0.258 to 0.361) as it is the case with their oxygen packing fractions (from 59.1 to 65.0%). However, for minerals with different formulae, the Δ*V*_norm_/OPF correlation is not intuitively understandable. Δ*V*_norm_ decreases generally with an increase of the number of atoms in the formula unit. It may be that the increase of the number of different elements and different polyhedra in the structure increases the probability that the mineral becomes less dense. However, the data of melilite should also be mentioned in this context. Melilite´s formula has neither a large nor a small number of elements. Nevertheless, the Mg-Al substitution in melilite is characterised by a very low strain (Δ*V*_norm_ = 0.042), which is certainly a consequence of its open packed structure (OPF = 49.9%). The oxygen packing fraction may, therefore, be the dominant factor for determining Δ*V*_norm_. 

The coordination number of the substituted cation does not always have the same value within a certain substitution and is quite heterogenous for the Mg-Ca substitution. The Ca site in different minerals increases from 6 to 12 with a simultaneous increase of OPF. These circumstances may result in a flattening of the *w*^H^_MgCa_/Δ*V*_norm_ and *w*^H^_MgCa_/OPF correlations. In MgSiO_3_ perovskite, as another example, Si(Al) has not tetrahedral but octahedral coordination. This might be a reason for producing the perovskite outlier in the *w*^H^/OPF correlation. On the other hand, melilite has a tetrahedral Mg(Al)-site and plots on the Mg-Al line, where all other minerals have Mg(Al) in octahedral coordination. Melilite´s *w*^H^_MgAl_ is slightly negative, which is a surprising observation, because cation substitutions are thought to produce zero (substitutions without local strain heterogeneities) or positive Δ*H*^mix^ in the case that local strain heterogeneities exist (Tarantino et al. 2003). However, the generated strain for the Mg-Al substitution in melilite is small (Δ*V*_norm_ = 0.042).

## Conclusions

It was shown in this study that the interaction energies of some substitutions strongly depend on the oxygen packing fraction, i.e., the Mg-Al and Mg-Ti exchanges and that of the Si-Al in chain and sheet silicates. This dependence can be understood as follows: The denser the oxygen packing, the stronger the impact of the local strain heterogeneities – caused by a substituted cation – will be on the whole unit cell. The strain heterogeneities of the Si-Al substitutions in island silicates may be absorbed by the less rigid neighbouring polyhedra producing the flat *w*^H^/OPF correlation. On the other hand, the *w*^H^_MgCa_/OPF correlation may be superimposed by the increasing coordination numbers with increasing OPF, flattening this correlation.

There is also a *w*^H^/Δ*V*_norm_ correlation, which is, however, not easily applicable to substitutions involving cations with different charges because the volume of the charged end members is only accessible by DFT methods.

The calculated *w*^H^ parameters represent the interaction energies between elements on a single crystallographic site. However, the degree of local charge balances and the degree of cation ordering influence the macroscopic interaction energies as demonstrated by DFT calculations (Dachs and Benisek 2019; Benisek and Dachs 2020). Other theoretical studies have also shown that Δ*H*^mix^ increases with temperature because of such effects, as demonstrated for garnets, diopside-jadeite solid solution, carbonites, and halides (e.g., Vinograd and Sluiter 2006; Vinograd et al. 2007, 2009; Vinograd and Winkler 2010). If one end-member is involved in the disordering processes as is the case with Tschermak´s substituted pyroxene and melilite, Δ*H*^mix^ can decrease with temperature (Sack and Ghiorso 2017; Sack 2021).

The interaction energy of the investigated Mg-Al substitution increases from minus 32.5 kJ/mol (melilite) to a huge value of plus 516.5 kJ/mol (spinel). A neglection of this dependence must result in inadequate thermodynamic models. The use of OPF-dependent interaction energies for substitutions that are characterised by large strains and the formulation of appropriate models for cation ordering should contribute to more reliable future mixing models.

## Supplementary Information

Below is the link to the electronic supplementary material.

Supplementary file1 (PDF 223 KB)

## Data Availability

All available data are given in the text and supplementary material.
