# Supplementary material for: The packing fraction of the oxygen sublattice: its impact on the heat of mixing
Source: Phys Chem Miner. 2024 Jun 4;51(3):23. doi: 10.1007/s00269-024-01277-6 (PMC11150184; doi:10.1007/s00269-024-01277-6)
Supplement: Supplementary file 1 — Supplementary file1 (PDF 223 KB) [file 269_2024_1277_MOESM1_ESM.pdf]

Supplementary material from the study “The packing fraction of the oxygen sublattice: Its impact on the heat of mixing” published by Artur Benisek and Edgar Dachs in the “Physics and Chemistry of Minerals”.

Tab. 1 The enthalpic interaction parameter (w^H) as a function of the oxygen packing fraction (OPF) and the normalised volume difference ($(V_2-V_1)/V_2$) for different substitutions in different minerals.
w^H was obtained by fitting ΔH^{mix} data, i.e., $\Delta H^{mix} = H_{AB} - (X_A H_A + X_B H_B)$, using the following equation:
$\Delta H^{mix} = X_A X_B w^H$.
X_A, X_B and H_A, H_B represent the mole fractions and the enthalpies of the A and B component, respectively. H_{AB} is the enthalpy of the solid solution at a given composition and ΔH^{mix} is the enthalpy of mixing.
For application reasons, the Mg-end members were always used to calculate OPF. For comparison reasons, w^H was normalised to the exchange of one mole of cations (the result for cordierite, as an example, was divided by 2 because 2 Mg per formula unit were substituted).

Substitution	Minerals	End members	Mixing on site(s)	OPF (in %)	$(V_2-V_1)/V_2$	w^H (kJ/mol)
Mg – Al	Melilite	$Ca_2(Mg)^{T1}Si_2O_7$<br>$\{Ca_2(Al)^{T1}Si_2O_7\}^{1+}$	1 T1	48.9	0.042	-32.5
	Chlorite	$(Mg)^{M1}(Mg)_2^{M2}(Mg)_2^{M3}(Mg)^{M4}Si_4O_{10}(OH)_8$<br>$\{(Mg)^{M1}(Mg)_2^{M2}(Mg)_2^{M3}(Al)^{M4}Si_4O_{10}(OH)_8\}^{1+}$	1 M4	52.5	0.104	29.3
	Biotite	$K(Mg)^{M1}(Mg)_2^{M2}[(OH)_2AlSi_3O_{10}]$<br>$\{K(Al)^{M1}(Mg)_2^{M2}[(OH)_2AlSi_3O_{10}]\}^{1+}$	1 M1	52.4	0.128	82.5
	Cordierite	$(Mg)_2^{M}Al_4Si_5O_{18}\ H_2O$<br>$\{(Al)_2^{M}Al_4Si_5O_{18}\ H_2O\}^{2+}$	2 M	52.0	0.123	131.4
	Amphibole	$(Ca)_2^{M4}(Mg)_2^{M1}(Mg)_2^{M2}(Mg)^{M3}[(OH)_2Si_8O_{22}]$<br>$\{(Ca)_2^{M4}(Mg)_2^{M1}(Al)_2^{M2}(Mg)^{M3}[(OH)_2Si_8O_{22}]\}^{2+}$	2 M2	56.0	0.126	141.1
	Talc	$(Mg)^{M1}(Mg)_2^{M2}[(OH)_2Si_4O_{10}]$<br>$\{(Al)^{M1}(Mg)_2^{M2}[(OH)_2Si_4O_{10}]\}^{1+}$	1 M1	56.3	0.185	179.2
	Pseudo-brookite	$(Mg)Ti_2O_5$<br>$\{(Al)Ti_2O_5\}^{1+}$	1 M	58.5	0.192	207.8
	Pyroxene	$(Ca)^{M2}(Mg)^{M1}Si_2O_6$<br>$\{(Ca)^{M2}(Al)^{M1}Si_2O_6\}^{1+}$	1 M1	57.9	0.201	213.6
	Sulphate 4 x hydrated	$(Mg)SO_4 \cdot 4\ H_2O$<br>$\{(Al)SO_4 \cdot 4\ H_2O\}^{1+}$	1 M	56.5	0.260	236.3
	Olivine	$(Mg)^{M1}(Mg)^{M2}SiO_4$<br>$\{(Al)^{M1}(Mg)^{M2}SiO_4\}^{1+}$	1 M1	57.9	0.258	275.6
	Wadsleyite	$(Mg)_{0.5}^{M1}(Mg)_{0.5}^{M2}(Mg)^{M3}SiO_4$<br>$\{(Al)_{0.5}^{M1}(Al)_{0.5}^{M2}(Mg)^{M3}SiO_4\}^{+1}$	1 (M1 + M2)	62.1	0.232	285.0
	Sulphate 1 x hydrated	$(Mg)SO_4 \cdot H_2O$<br>$\{(Al)SO_4 \cdot H_2O\}^{1+}$	1 M	60.3	0.326	350.5
	Ilmenite	$(Mg)^{M}TiO_3$<br>$\{(Al)^{M}TiO_3\}^{1+}$	1 M	62.0	0.303	406.8
	Tungstate	$(Mg)WO_4$<br>$\{(Al)WO_4\}^{1+}$	1 M	62.6	0.319	446.4
	Sulphate anhydrous	$(Mg)SO_4$<br>$\{(Al)SO_4\}^{1+}$	1 M	64.0	0.375	477.0
	Spinel	$(Mg)_2^{M}SiO_4$<br>$\{(Al)_2^{M}SiO_4\}^{2+}$	2 M	63.7	0.361	516.5

Si – Al	Biotite	$KMg_3[(OH)_2(Al, Si)^{T1}(Si)_2^{T2}O_{10}]$<br>$\{KMg_3[(OH)_2(Al)_2^{T1}(Si)_2^{T2}O_{10}]\}^{1-}$	2 T1<br>(Al-avoid)	52.4	0.156	47.8
	Amphibole	$Ca_2Mg_5[(OH)_2(Si)_4^{T1}(Si)_4^{T2}O_{22}]$<br>$\{Ca_2Mg_5[(OH)_2(Al, Si_3)^{T1}(Si)_4^{T2}O_{22}]\}^{1-}$	4 T1	56.0	0.212	136.1
	Pyroxene	$CaMg(Si)_2^{T}O_6$<br>$\{CaMg(Al)^{T1}(Si)^{T2}O_6\}^{1-}$	1 T 1<br>(Al-avoid)	57.9	0.364	285.6
	perovskite	orthorhombic $Mg(Si)^{B}O_3$<br>orthorhombic $\{Mg(Al)^{B}O_3\}^{1-}$	1 B	77.1	0.266	577.4
Si – Al island, double island	Melilite	$Ca_2Mg(Si)_2O_7$<br>$\{Ca_2Al(Al)(Si)O_7\}^{1-}$	1 T2a	48.9	0.167	148.1
	Olivine	$Mg_2(Si)^{T}O_4$<br>$\{Mg_2(Al)^{T}O_4\}^{1-}$	1 T	57.9	0.190	172.0
	Wadsleyite	$Mg_2(Si)^{T}O_4$<br>$\{Mg_2(Al)^{T}O_4\}^{1-}$	1 T	62.1	0.182	176.1
	Spinel	$Mg_2(Si)^{T}O_4$<br>$\{Mg_2(Al)^{T}O_4\}^{1-}$	1 T	63.7	0.182	186.5
Mg – Ti^{4+}	Biotite	$K(Mg)^{M1}(Mg)_2^{M2}[(OH)_2AlSi_3O_{10}]$<br>$\{K(Ti)^{M1}(Mg)_2^{M2}[(OH)_2AlSi_3O_{10}]\}^{2+}$	1 M1	52.4	0.179	384.7
	Pyroxene	$(Ca)^{M2}(Mg)^{M1}Si_2O_6$<br>$\{(Ca)^{M2}(Ti)^{M1}Si_2O_6\}^{2+}$	1 M1	57.9	0.305	877.3
	Olivine	$(Mg)^{M1}(Mg)^{M2}SiO_4$<br>$\{(Mg)^{M1}(Ti)^{M2}SiO_4\}^{2+}$	1 M1	57.9	0.402	1168.6
	Spinel	$(Mg)_2^{M}SiO_4$<br>$\{(Ti)_2^{M}SiO_4\}^{4+}$	2 M	63.7	0.405	1732.5
Mg – Ca	Hydroxide	$(Mg)(OH)_2$<br>$(Ca)(OH)_2$	1 M	54.9	0.24	50.4
	Oxide	$(Mg)O$<br>$(Ca)O$	1 M	55.8	0.303	76.0
	Amphibole	$(Mg)_2^{M4}(Mg)_2^{M1}(Mg)_2^{M2}(Mg)^{M3}[(OH)_2Si_8O_{22}]$<br>$(Ca)_2^{M4}(Mg)_2^{M1}(Mg)_2^{M2}(Mg)^{M3}[(OH)_2Si_8O_{22}]$	2 M4	57.0	0.017	24.4
	Olivine	$(Mg)^{M1}(Mg)^{M2}SiO_4$<br>$(Mg)^{M1}(Ca)^{M2}SiO_4$	1 M2	57.9	0.139	39.9
	Pyroxene	$(Mg)^{M2}(Mg)^{M1}Si_2O_6$<br>$(Ca)^{M2}(Mg)^{M1}Si_2O_6$	1 M2	60.7	0.074	65.3
	Carbonate	$(Mg)CO_3$<br>$(Ca)CO_3$	1 M	68.1	0.228	34.1
	Garnet	$(Mg)_3^{X}Al_2Si_3O_{12}$<br>$(Ca)_3^{X}Al_2Si_3O_{12}$	3 X	68.9	0.091	11.8
	Perovskite	orthorhombic $(Mg)^{A}SiO_3$<br>cubic $(Ca)^{A}SiO_3$	1 A	77.1	0.052	76.4
Mg – Fe^{2+}	Biotite	$K(Mg)_3^{M}[(OH)_2AlSi_3O_{10}]$<br>$K(Fe)_3^{M}[(OH)_2AlSi_3O_{10}]$	3 M	52.4	0.01	-2.2
	Brucite	$(Mg)(OH)_2$<br>$(Fe)(OH)_2$	1 M	53.9	0.01	-3.5
	Pyroxene	$(Ca)^{M2}(Mg)^{M1}Si_2O_6$<br>$(Ca)^{M2}(Fe)^{M1}Si_2O_6$	1 M1	57.9	0.01	1.0
	Olivine	$(Mg)_2^{M}SiO_4$<br>$(Fe)_2^{M}SiO_4$	2 M	57.9	0.02	-3.5
	Spinel	$(Mg)_2^{M}SiO_4$<br>$(Fe)_2^{M}SiO_4$	2 M	63.7	0.03	-24.4
	Garnet	$(Mg)_3^{X}Al_2Si_3O_{12}$<br>$(Fe)_3^{X}Al_2Si_3O_{12}$	3 X	68.9	0.03	-3.2
	Perovskite	orthorhombic $(Mg)^{M}SiO_3$<br>orthorhombic $(Fe)^{M}SiO_3$	1 M	77.1	0.02	-2.3

Tab. 2 Fit parameter for the interaction parameter (w^H) as a function of oxygen packing fraction (OPF) in %.

Substitution	w^H (kJ/mol)	R^2
Mg – Al	-1680.9 + 33.4*OPF	0.91
Si – Al	-2116.7 + 41.0*OPF	0.90
Si – Al for island, double island	30.7 + 2.41*OPF	0.97
Mg – Ti	-5899.5 + 119.7*OPF	0.95
Mg – Ca	36.0 + 0.17*OPF	n.a.
Mg – Fe	1.2 - 0.1*OPF	n.a.

Tab. 3 Fit parameter for the interaction parameter (w^H) as a function of normalised volume difference ($(V_2-V_1)/V_2$).

Substitution	w^H (kJ/mol)	R^2
Mg – Al	-99.3 + 1566.1*$(V_2-V_1)/V_2$	0.95
Mg – Ti	-518.4 + 4830.9*$(V_2-V_1)/V_2$	0.83
Si – Al	-100.3 + 1047.1*$(V_2-V_1)/V_2$	0.99
Mg – Ca	33.64 + 97.32*$(V_2-V_1)/V_2$	n.a.
Mg – Fe	n.a.	n.a.

Tab. 4 Fit parameter for the normalised volume difference ($(V_2-V_1)/V_2$) as a function of oxygen packing fraction (OPF) in %.

Substitution	$(V_2-V_1)/V_2$	R^2
Mg – Al	-0.94 + 0.02*OPF	0.95
Mg – Ti	-0.83 + 0.02*OPF	0.74
Si – Al	-0.84 + 0.02*OPF	0.95

Tab. 5 Comparison of the enthalpic interaction parameters (W^H) in kJ mol^{-1} for different Mg-Ca substitutions with literature values, defined as $\Delta H^{mix} = X_A X_B W$. It was normalised to the exchange of one mole of cations. In the case that the literature mixing model used asymmetric formalism, the two values were averaged for comparison reasons.

	This study	Literature
MgO – CaO	76.0	87.4 [1)]
Mg_2SiO_4 – $CaMgSiO_4$	39.9	48.4 [2)]
$MgCO_3$ – $CaCO_3$	34.1	27.8 [3)]
$Mg_3Al_2Si_3O_{12}$ – $Ca_3Al_2Si_3O_{12}$	11.8	12.2 [4)]

[1)] Liang and Schmid-Fetzer 2018
[2)] Kawasaki 1998
[3)] Vinograd et al. 2009
[4)] Newton et al. 1977
